# A Case of Malignant Brenner Tumor With High FDG Uptake

**DOI:** 10.1002/ccr3.72687

**Published:** 2026-05-29

**Authors:** Jie Li, Longdong Fang, Tian‐yun Wang, Jin‐gang Hao

**Affiliations:** ^1^ Department of PET/CT Center The Second Affiliated Hospital of Kunming Medical University Kunming China; ^2^ Department of Pathology C The Second Affiliated Hospital of Kunming Medical University Kunming China

**Keywords:** ^18^F‐FDG PET/CT, diagnosis, high FDG uptake, malignant Brenner tumor

## Abstract

Malignant Brenner Tumor (MBT) is a rare malignant neoplasm originating from the ovarian surface epithelium. This type of tumor typically lacks specific clinical manifestations and dedicated tumor markers. In our case, we present a complete set of clinical information, diagnosis and treatment protocols, and follow‐up results of one case of MBT. We highlight the crucial role of ^18^F‐FDG PET/CT in the diagnosis and treatment process, which significantly improves the diagnostic accuracy for distinguishing benign from malignant tumors and predicts tumor invasiveness. Meanwhile, our case exhibits the highest FDG uptake among all reported cases to date, thereby supplementing and deepening our understanding of the FDG metabolic characteristics of MBT.

## Key Clinical Message

Malignant Brenner Tumor is a rare malignant neoplasm originating from the ovarian surface epithelium. ^18^F‐FDG PET/CT significantly improves the accuracy of differentiating benign and malignant tumors, predicts tumor aggressiveness, and aids treatment planning. Moreover, our case exhibited the highest FDG uptake among all reported cases.

## 1 Introduction

Brenner tumors originate from the ovarian surface epithelium, accounting for 1% to 2% of ovarian epithelial tumors [1]. According to the WHO classification, they are categorized into benign, borderline, and malignant types. The majority of Brenner tumors are benign, while the malignant subtype is extremely rare, with an incidence of less than 5% [2]. Malignant Brenner tumor (MBT) predominantly affects women aged 50 to 70 years. Notably, the risk of malignancy in Brenner tumors increases with advancing patient age [3]. Characterized by nonspecific clinical manifestations, MBT typically presents with abdominal pain, abdominal distension, or a pelvic mass. To date, no specific tumor markers for Brenner tumors have been identified [4].

However, reports indicate that carbohydrate antigen 125 (CA125) levels are elevated in patients with MBT, and these elevated levels tend to decrease following treatment [3]. MRI also holds diagnostic value, enabling a clearer assessment of whether lesions exhibit cystic changes, hemorrhage, necrosis, or fatty components. Nevertheless, MRI lacks specific characteristic signs for definitive diagnosis. MBT demonstrates ^18^F‐fluorodeoxyglucose (^18^F‐FDG) uptake, with the extent of uptake directly correlated with the degree of tumor malignancy. Positron emission tomography combined with computed tomography (PET/CT) enhanced the visualization of the tumor's malignant potential, providing valuable insights into its aggressive characteristics [5].

We present a case of a 51‐year‐old female diagnosed with MBT, whose clinical staging was accurately determined via PET/CT imaging. This case report has been prepared in accordance with the “Ovarian cancer staging and follow‐up: updated guidelines from the European Society of Urogenital Radiology female pelvic imaging working group” [6].

## 2 Case History and Examination

A 51‐year‐old female presented with lower abdominal pain without obvious predisposing factors one month prior to admission. She underwent menopause at the age of 43, and there had been no vaginal bleeding or discharge since then. No family history of disease and no denial of past medical history.

## 3 Methods (Differential Diagnosis, Investigations and Treatment)

Tumor marker tests revealed that CA125 was 601.82 U/mL (Reference range < 35.0 U/mL), CA15‐3 was 72.12 U/mL (Reference range < 31.0 U/mL), CA19‐9 was 22.4 U/mL (reference range: < 37.00 U/mL), CA72‐4 was 3.19 U/mL (reference range: < 6.90 U/mL), and β‐HCG was 1.97 IU/L (reference range: < 5.00 IU/L). Ultrasonography revealed a hyperechoic, heterogeneous solid mass in the right adnexal area, classified as O‐RADS 5. Her MRI scan showed a mass in the right adnexal region of the pelvis, displaying iso‐intense T1 and slightly hyperintense T2 signals. The mass measured approximately 6.1 cm × 6.0 cm × 5.4 cm and exhibited mild enhancement on contrast‐enhanced scanning (Figures 1, 2, 3). ^18^F‐FDG‐PET/CT imaging demonstrated a space‐occupying lesion in the right pelvic region with increased FDG uptake, and the maximum standardized uptake value (SUVmax) was approximately 18.2 (Figures 4 and 5). No other lesions with abnormally increased FDG metabolism were found. A preliminary diagnosis of malignant tumor of the right ovary was established in this case; FIGO stage: IIA. The differential diagnosis of this case includes benign ovarian tumors, pelvic inflammatory masses, endometriotic cysts and tuberculous pelvic inflammation. Malignant tumors typically exhibit ill‐defined borders, greater invasiveness, and higher FDG metabolism. Clinical symptoms such as fever are usually not obvious, and the lesions show no significant improvement following anti‐inflammatory therapy. The patient underwent a total hysterectomy along with bilateral oophorectomy and fallopian tube removal, as well as a mesentery resection and lymph node dissection in our hospital. Pathological examination of HE‐stained sections revealed diffuse infiltration of tumor cells, accompanied by numerous bizarre nuclei, a high nuclear‐cytoplasmic ratio, and frequent mitotic figures. Immunohistochemical staining showed positive results for GATA3 (+), P63 (+), P16 (+), mutant P53, and Ki‐67 (80%) (Figure 6).

**FIGURE 1:**
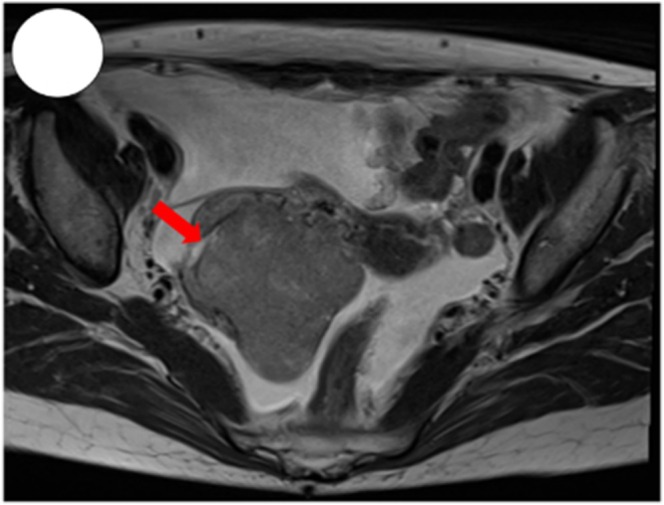
Isointense signal on T1WI.

**FIGURE 2:**
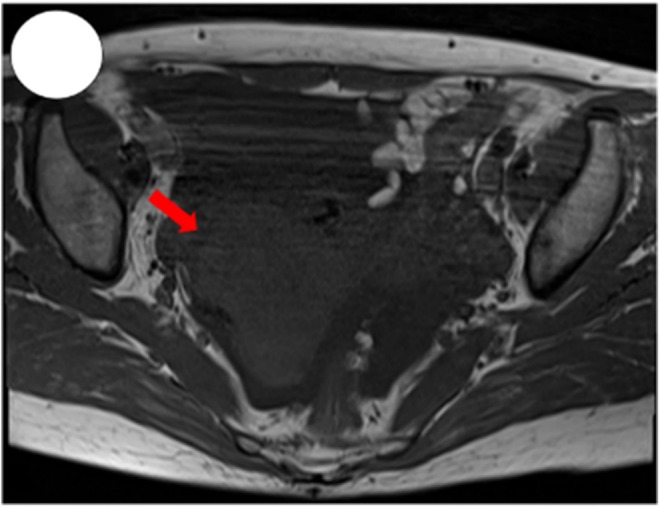
Slightly hyperintense signal on T2WI.

**FIGURE 3:**
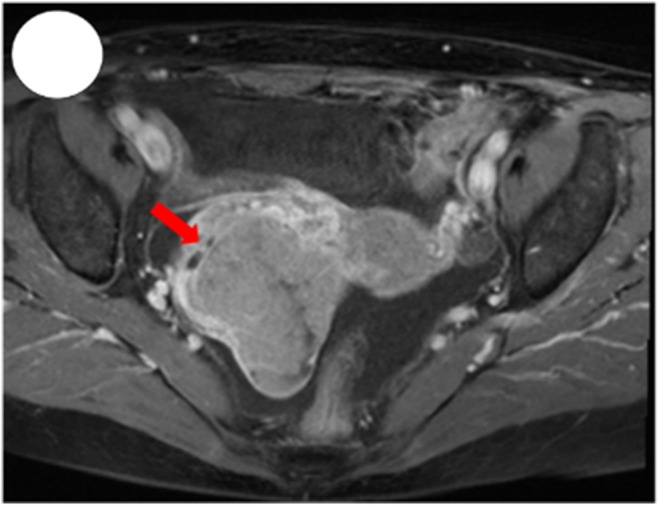
Mild enhancement observed on contrast‐enhanced MRI scan.

**FIGURE 4:**
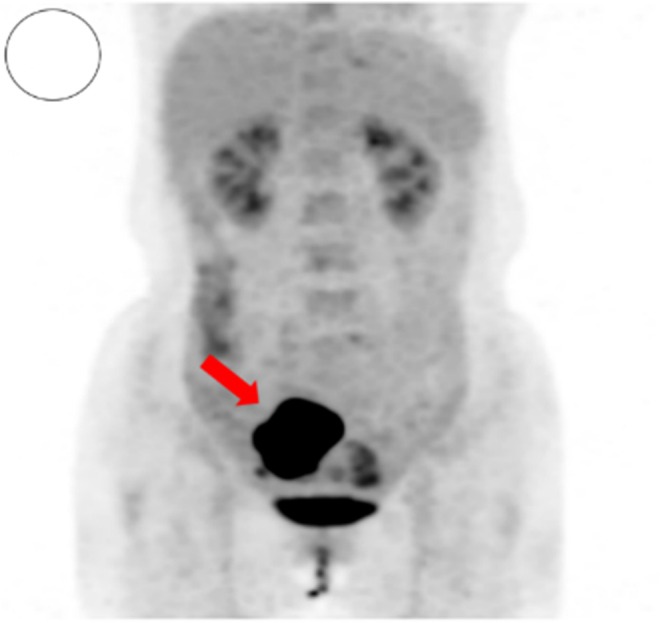
The MIP image from the PET scan reveals a lesion in the right pelvic region with abnormally elevated FDG metabolism.

**FIGURE 5:**
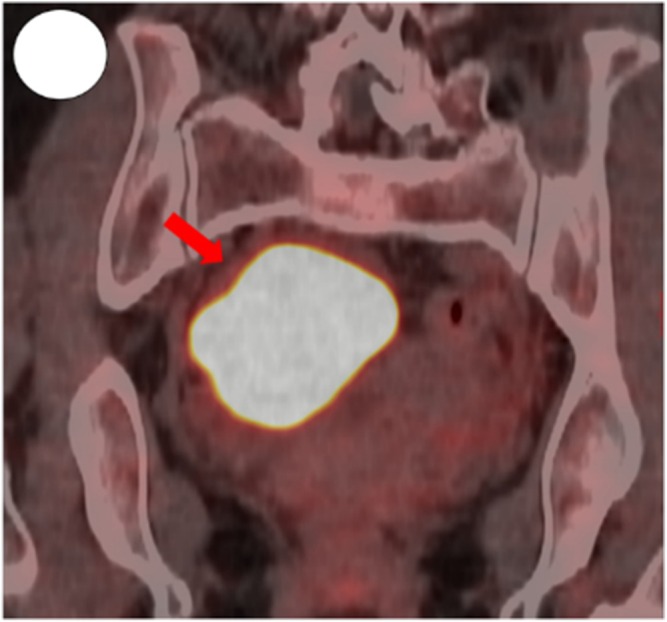
The coronal view of PET/CT shows a space‐occupying lesion in the pelvic cavity with increased FDG uptake, and the SUVmax is approximately 18.2.

**FIGURE 6:**
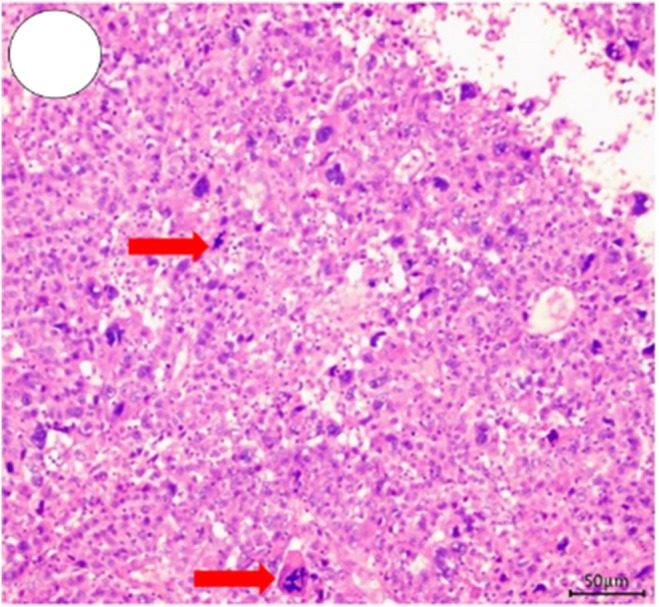
High magnification (×200) reveals numerous bizarrely nucleated cells with a high nuclear‐to‐cytoplasmic ratio. Mitotic figures are readily apparent (as indicated by arrows). The atypical cells exhibit polygonal and horseshoe‐shaped morphologies.

## 4 Conclusion and Results (Outcome and Follow‐Up)

The pathological diagnosis was Malignant Brenner tumor of the ovary with transitional epithelial differentiation and poorly differentiated carcinoma components and no metastatic involvement was detected in the resected lymph nodes (0/15). Subsequently, the patient underwent six cycles of chemotherapy with the regimen of paclitaxel (240 mg) and carboplatin (600 mg). The assessment was conducted after the treatment was carried out at the end of the last cycle. No abnormal lesions were found through CT and MRI examinations. This patient achieved complete remission (CR).

## 5 Discussion

The patient in this case presented with right lower abdominal pain as the main clinical manifestation, without other special symptoms [7, 8]. Elevated levels of tumor markers CA125 and CA15‐3 suggested the possibility of reproductive system‐related lesions. Transvaginal ultrasound examination and MRI examinations both indicated a tumor in the right adnexal region, which was suspected to be malignant. It is worth noting that the MRI enhancement degree of MBT is significantly higher than that of benign and borderline Brenner tumors [8]. Combined with the above conventional examinations, the preliminary diagnosis of the disease can be completed. To further clarify the benignity or malignancy of the tumor, evaluate its invasiveness, determine the stage, and formulate a scientific and reasonable clinical treatment plan, we performed a PET/CT examination for the patient. In ^18^F‐FDG‐PET/CT imaging, the FDG uptake of benign and borderline Brenner tumors was low or slightly increased, while the FDG uptake of MBT was significantly increased [9, 10, 11]. This indicating that ^18^F‐FDG‐PET/CT plays a key role in differentiating benign, borderline and malignant Brenner tumors. Using an SUVmax threshold of 2.9 as the detection standard for malignant tumors, the analysis results showed that the sensitivity, specificity, positive predictive value (PPV), and negative predictive value (NPV) of this standard were 80.6%, 94.6%, 91.5%, and 87.1%, respectively [12]. In this case, the tumor showed significantly increased FDG uptake with an SUVmax of approximately 18.2, which could be clearly diagnosed as a malignant tumor according to the above standard. Meanwhile, whole‐body PET/CT imaging did not find any lesions invading adjacent tissues or distant metastases, providing an important basis for clinical staging. PET/CT is capable of confirming MBT as FIGO StageIIA. Clinical studies have shown that there is a positive correlation between tumor FDG uptake level and invasiveness; the higher the uptake, the stronger the tumor invasiveness and the poorer the differentiation. The immunohistochemical result of the tumor in this case showed a Ki‐67 index of 80%, which was consistent with the high FDG uptake, further confirming this conclusion. The clinical manifestations and conventional auxiliary examinations of ovarian Brenner tumors lack obvious specificity, which is prone to missed diagnosis or misdiagnosis [8]. However, PET/CT examination can significantly improve the accuracy of diagnosing the benignity or malignancy of the tumor and effectively predict the invasiveness of malignant tumors. In addition, this examination can comprehensively evaluate the lesions, providing reliable imaging evidence for clinicians to formulate treatment plans, expand the surgical resection range of lesions, perform adjuvant chemotherapy, and evaluate the curative effect after treatment.

In conclusion, the ^18^F‐FDG‐PET/CT examination can effectively assist in differentiating the benign and malignant nature of ovarian Brenner tumors and predicting the invasiveness of the tumors. It provides important support for clinical diagnosis and the formulation of treatment plans and has high clinical application value.

## Author Contributions

**Jie Li:** conceptualization, writing – original draft. **Longdong Fang:** resources, software. **Tian‐yun Wang:** validation, visualization. **Jin‐gang Hao:** conceptualization, writing – review and editing.

## Funding

The Basic Research Project Fund of Kunming Medical University, Yunnan Provincial Department of Science and Technology, China (202401AY070001‐337).

## Disclosure

The views expressed in this report are solely those of the author(s) and do not represent the official positions of any affiliated institutions.

## Ethics Statement

The authors have nothing to report.

## Consent

The authors confirm that written informed consent was obtained from the patient for publication of this case report and accompanying clinical data.

## Conflicts of Interest

The authors declare no conflicts of interest.

## Data Availability

Data sharing not applicable to this article as no datasets were generated or analysed during the current study.
